# Attitude to the Menopause and Sex amongst Middle-Aged Women in a Family Medicine Clinic in Ibadan, Nigeria

**DOI:** 10.1155/2016/2031056

**Published:** 2016-11-08

**Authors:** Folasade Adenike Bello, Olufunmilola Olutosin Daramola

**Affiliations:** ^1^Department of Obstetrics and Gynaecology, College of Medicine, University of Ibadan, Ibadan, Nigeria; ^2^Department of Family Medicine, University College Hospital, Ibadan, Nigeria

## Abstract

*Background*. Menopause is the expected end of reproductive life. Having a positive attitude towards it has been shown to result in a positive experience, while a negative attitude is associated with negative experiences and symptoms. Traditionally, women often abstain from sex after menopause. The study aimed to determine the level of awareness and perceptions about the menopause and sex in perimenopausal women attending a general outpatient clinic.* Methods*. Women over 40 years were recruited from the Family Medicine Department of University College Hospital, excluding those who were menopausal. Data analyses were done with chi-square test (*p* < 0.05).* Results*. Most (302; 86.4%) of the 352 surveyed participants were aware of the menopause. Only 36.1% anticipated associated symptoms. About half (55.7%) were indifferent to menopause onset, while 23% had a positive attitude and 21.4% had a negative attitude, respectively. Younger women were less likely to have a positive attitude to the menopause (*p* = 0.04). There were negative cultural beliefs towards sex. Sexual activity was low and declined with age (*p* < 0.001). Many women would like treatment to improve their sexual activity.* Conclusion*. Most participants had a favourable disposition towards the menopause, though sexual relationships suffer. Counselling and treatment should be offered.

## 1. Introduction

Menopause marks the end of the natural reproductive capacity of a woman. The clinical diagnosis is made retrospectively after twelve months of amenorrhoea. For many women, it is a welcome relief from menses and eliminates the risk of pregnancy [1, 2], while for others, in whom childbearing is still desired, menopause is not welcome [1]. Many women are aware that their menstrual periods will cease someday, but anecdotal evidence in our clinical settings shows that they are mostly not aware of the other changes and symptoms that accompany menopause. This makes them bewildered, stressed, and confused when they start to suffer from these. Women may think that these symptoms are ominous signs of illness or even voodoo attacks [3]. This is made worse as these symptoms usually occur before the cessation of menses, so sufferers do not readily associate them with the menopause.

Traditionally, Nigerian women often abstain from sexual intercourse after the menopause [1–3]. This may be partly due to climacteric symptoms like loss of libido and dyspareunia but is also largely influenced by cultural beliefs. Local beliefs include the notion that menstrual flow “cleanses” the woman, and, in the absence of menses, if she has sex, these impurities will cause illness in her [3].

Previous workers have concluded that having a positive attitude towards the menopause is often associated with having a positive experience with menopause, and having a negative attitude was associated with negative experiences and symptoms [4]. Assessing perception of menopause enables us to assess knowledge gaps and may guide health talks and counselling. When women have better knowledge of what to expect, they are more likely to understand and be accepting of their symptoms. Also, if they are aware that management options are available, they may also be more likely to seek health care if required.

First-line healthcare providers such as family physicians are in a position to empower women through appropriate counselling and education that is geared towards improving women's attitude towards the menopause and dispelling unhelpful or untruthful traditional beliefs. The assurances given to a woman that her symptoms are not a feature of a disease help to alleviate her fears. There is also the opportunity to enlighten women on health-related issues like nutrition, osteoporosis, cardiovascular disease, and menopausal weight gain.

The study aimed to determine the level of awareness and perceptions about the menopause and sex in perimenopausal women attending a general outpatient clinic.

## 2. Materials and Methods

The study utilized a cross-sectional, descriptive design. The setting was the Family Medicine Department of the University College Hospital, Ibadan, Nigeria. It is a referral hospital that serves southwest Nigeria. This clinic was chosen for the study, rather than the Gynaecology Department, as it is a primary care clinic and more likely to reflect the community. Also, the researchers would be less likely to recruit women with concurrent gynaecological problems from the Family Medicine Clinic.

Study subjects were women over 40 years who were yet to attain menopause, as being menopausal will influence their perceptions. Women who last had a menstrual period one year or more previously were excluded, as well as women who had debilitating illness.

Ethical approval was obtained from the joint University of Ibadan/University College Hospital Ethics Committee. Informed consent was obtained from participants before administration of questionnaires.

A structured closed- and open-ended questionnaire was used to obtain data. Social and demographic features were explanatory variables, while outcome variables were positive or negative attitude to menopause. Chi-square tests were done (*p* < 0.05). Data were analysed with IBM SPSS Statistics 20 software.

## 3. Results

The mean age of the respondents was 46.3 ± 4.0 years. They were all premenopausal and had attained menarche at averagely 14.2  ±  1.7 years. Their social and demographic characteristics were well distributed (Table 1). Most of them (256; 72.7%) had already started experiencing irregular periods.

Of the 352 women who were surveyed, 304 (86.4%) were aware of menopause (i.e., that menses would naturally cease at some point). They expected the menopause to occur between ages 41 and 65 yrs; most respondents expected this to happen at 50 years. Only 127 (36.1%) anticipated that symptoms or health changes would accompany the menopause; the rest expected no other changes. Eighty-four (23.9%) anticipated body weakness and pains and 20 (5.7%) expected “internal heat” and sweating, while 23 (6.5%) expected an improvement in their health status. The respondents' sources of information about the menopause included their health care providers (17.0%), books and other print media (12.2%), friends and peers (45.7%), aunts or older sisters (15.1%), TV or radio (12.2%), and others (1.4%). Only forty-eight (13.6%) report that their doctors had ever discussed the health risks associated with the menopause with them.

On attitude towards the menopause (Table 2), 75 (21.4%) had a negative attitude towards it: 52 felt it would make them incomplete as women, the other 23 were concerned that it would be followed by persistent ill health, and others still wanted to have children, while some felt they were too young to undergo menopause. Eighty-one (23.0%) looked forward to a welcome relief from menses, while most respondents were indifferent. Respondents volunteered their cultural beliefs about the menopause. The most common belief was that sexual intercourse after the menopause causes ill health to the woman (179; 50.9%). Fifty-two (14.8%) said it was part of the natural aging process of a woman; 38 (10.8%) suggested a belief that it marked the end of femininity and, thereafter, she “becomes a man”; 12 (3.4%) said that the menopause heralds the onset of persistent sickness and death in the woman. Ten (2.8%) women volunteered less popular beliefs: that menopause leads to erectile dysfunction in partners, that severe body pains in the menopause are due to male children being left unborn within the woman, and that the onset of menopause is hastened if a woman does not have regular sex. The respondents do not all have the prevalent cultural beliefs which they volunteered though: only 133 (37.8%) do.

Table 3 summarizes their perception of sexual intercourse. Respondents that reported less frequent intercourse gave many reasons for this. The most recurring ones were fear of disease, loss of libido, dyspareunia, cultural beliefs, and presence of younger cowives whom the husband could have coitus with. Only two women considered their husband as understanding of their diminished sexual interest or activity. When analysed against age group, women between 51 and 55 years of age were likely to have had their last sexual episode more than six months previously (*p* < 0.001). If there is available treatment which can improve sexual relationships, 124 (35.2%) women would be interested in it. Only 17 (4.8%) have been offered treatment options by their health care providers. Only 22 (6.3%) had heard of hormone replacement therapy.

Table 4 explores the association of attitude towards menopause with selected social and demographic characteristics. Only age group showed a significant association, with younger women being less likely to have a positive attitude towards it (*p* = 0.04).

## 4. Discussion 

Middle-aged women who had not attained menopause were the subjects of this study, so that their personal experience would not influence their attitude towards this life phase. As most of them had started experiencing menstrual irregularities, it can be inferred that they were already in the transition.

A large proportion of subjects were aware of the menopause, higher than a previous figure of 67% [2], implying that awareness has improved over time. Most women's idea of age of onset of menopause was in consonance with documented average figures (49–51 years) [1, 5]. However, many of them were not aware that there were symptoms and health changes that accompany the menopause. When women experience these symptoms without warning, they find them to be more troubling and debilitating than when anticipated. Those who knew were able to identify body aches (which are usually from arthritis or osteoporosis) and hot flushes, which are the most common menopausal symptoms reported from Nigeria [1, 6, 7]. Most of the information they had was obtained from nonmedical sources. In their African-American counterparts in the US, older females were also seen to be important sources of information [8]. Only a small fraction had been told the health risks of the menopause. Health education therefore needs to be directed at this study population.

A fairly even proportion of respondents had positive or negative attitudes towards the menopause. A previous survey on a similar population showed that women mostly had a positive attitude towards menopause [2]. In the index study, some of the negative attitudes were influenced by cultural norms, but others were legitimate concerns, like those who still desired to bear children. There were mostly no features identified to be significantly associated with attitudes to menopause. Younger perimenopausal women were less accepting, but this seems to adjust with age, so it is probably a self-limiting factor. The large proportion of respondents who indicated indifference affirm that this life phase was largely acceptable to them.

The notion that postmenopausal sex causes ill health appears to be well established, as other studies have shown [1, 3]. These cultures are deep-seated, as they have not changed in decades [2, 9]. This may unfortunately encourage infidelity on the part of husbands who have sexual desires but are unwilling to cause their wives medical problems. Irrespective of whether respondents shared the cultural belief about sex, they most had a negative attitude to it. Most of them expressed their lack of interest in sex and that their sexual frequency had diminished, which seemed directly proportional to the women's age. Only two women consider their husbands as understanding with regard to this, inferring that this affects their partners (and likely their relationships) adversely. A sizeable proportion of the study group are open to using treatment methods directed at improving sex for them, indicating a willingness to adjust. Sexual dysfunction may be related to the menopause, even in Western cultures where cessation of sex is not the norm, or expected. As no validated tool was used in this study to assess sexual dysfunction, the differentiation may be limited. A review of 42 global studies showed that ethnic and cultural factors had an appreciable influence on the determination of sexual dysfunction in the menopause [10].

The lack of well-woman services may limit the opportunities to counsel women about the menopause. It may be difficult to factor general health talks into medical consultations where woman are presenting with widely varied complaints. A primary care or family medicine clinic such as this study's setting may be a plausible platform for educational materials and talks, as most patients are less likely to be really sick. The family physician is in a position to empower women through appropriate counselling and education that is geared towards dispelling traditional myths and taboos including the practice of sexual abstinence at menopause. The assurances given to a woman that her symptoms are not a feature of a disease will help to alleviate her fears. There is also the opportunity to enlighten women on other menopausal health-related issues like weight gain, cancer risks and screening, osteoporosis, and cardiovascular disease. Anticipatory guidance and care that can ameliorate the severity of these diseases can also be offered.

The onus may be on health care providers to initiate counselling on menopause and its symptoms with patients, to reassure them and to identify those that may benefit from available management options. It is not surprising that most respondents had not heard about hormone replacement therapy, as it is not readily available in the study location. The safety of HRT has been questioned based on evidence from clinical trials [11, 12] and even in the Western world; its use involves weighing its benefits against risks in each individual. Available alternative treatment, including natural supplements, can also be tailored to patients' individual needs.

## 5. Conclusion

In conclusion, most respondents are aware of the menopause and are mostly indifferent to it. Positive versus negative attitudes towards it are nearly equal. Sexual relationships suffer in the perimenopause. Counselling and treatment should be offered to these women to improve their sexual and family health and to dissipate fallacious cultural beliefs that adversely affect them.

## Figures and Tables

**Table 1:** Sociodemographic characteristics of study respondents.

Characteristic	*N* = 352 (%)
*Age group*	
41–45	181 (51.4)
46–50	114 (32.4)
51–55	57 (16.2)
*Marital status*	
Unmarried^*∗*^	65 (18.5)
Married	287 (81.5)
*Children*	
Have children	346 (98.3)
No currently living children	6 (1.7)
*Religion*	
Christianity	201 (57.1)
Islam	149 (42.3)
Traditional	2 (0.6)
*Tribe*	
Yoruba	283 (80.4)
Igbo	19 (5.4)
Hausa	41 (11.6)
Other tribes	9 (2.6)
*Education*	
None/informal	68 (19.3)
Primary	94 (26.7)
Secondary	64 (18.2)
Tertiary	126 (35.8)
*Occupation*	
Unemployed	10 (2.8)
Unskilled	65 (18.5)
Traders	163 (46.3)
Skilled	97 (27.6)
Professionals	17 (4.8)

^*∗*^including single, separated, divorced, or widowed respondents.

**Table 2:** Respondents' attitude to the menopause.

Attitude	*N* (%)
*Positive attitude*	
Welcome relief from menses	81 (23.0)
*Negative attitude*	
Will make them feel incomplete as women	52 (14.8)
They expect it to herald the onset of persistent ill health	11 (3.1)
Felt they were too young for this	6 (1.7)
Desire more children	6 (1.7)
*Indifferent attitude*	196 (55.7)

**Table 3:** Perimenopausal women's perception and practice of coitus.

*Attitude towards coitus*	*N* = 343
Interested in having sex	21 (6.0)
No interest in sex	247 (72.0)
Indifferent to sex	75 (21.9)

*Frequency of coitus in the past year*	*N* = 340
Decreased	173 (49.1)
Increased	15 (4.3)
No change	85 (24.1)
None in one year	67 (19.0)

*Last sexual exposure*	*N* = 338
Within 1 week	110 (32.5)
1–4 weeks	66 (19.5)
1–6 months	48 (14.2)
>6 months	114 (33.7)

*Perceived effect of reduced coital activity on partner*	*N* = 220
No effect noticed	94 (26.7)
Indifference (partner is not interested)	50 (14.2)
Causes quarrels	42 (11.9)
Partner is resigned	32 (9.1)
He understands	2 (0.6)

**Table 4:** Possible associations of positive and negative attitude to the menopause.

Characteristic	Positive attitude	Negative attitude	*p*
*Age group*			
41–45	38 (43.2)	50 (56.8)	
46–50	26 (60.5)	17 (39.5)	0.04
51–55	17 (68.0)	8 (32.0)	
*Marital status*			
Unmarried	15 (57.7)	11 (42.3)	0.52
Married	66 (50.8)	64 (492)	
*Type of marriage*			
Monogamous	53 (52.0)	49 (48.0)	>0.99
Polygamous	26 (52.0)	24 (48.0)	
*Has children*			
Yes	81 (52.9)	72 (47.1)	0.11^*∗*^
No	0 (0)	3 (100.0)	
*Level of education*			
Primary or less	31 (47.7)	34 (52.3)	0.37
Secondary or higher	50 (54.9)	41 (45.1)	
*Religion *			
Christianity	57 (55.3)	46 (44.7)	0.23
Islam	24 (45.3)	29 (54.7)	
*Menses has become irregular*			
Yes	59 (52.2)	54 (47.8)	0.99
No	22 (52.4)	20 (47.6)	
*Anticipation of unhealthy changes with the menopause*			
Yes	34 (49.3)	35 (50.7)	0.56
No	47 (54.0)	40 (46.0)	
*Frequency of sex in the past year*			
Increased activity	5 (55.6)	4 (44.4)	0.89^*∗*^
Decreased activity	43 (53.8)	37 (46.2)	
The same as usual	15 (46.9)	17 (53.1)	
None at all	18 (56.2)	14 (43.8)	

^*∗*^Fisher's exact test.
